# Supplementary material for: Preoptic leptin signaling modulates energy balance independent of body temperature regulation
Source: eLife. 2018 May 15;7:e33505. doi: 10.7554/eLife.33505 (PMC5953538; doi:10.7554/eLife.33505)
Supplement: Transparent reporting form [file elife-33505-transrepform.docx]

***eLife’s* transparent reporting form**

We encourage authors to provide detailed information *within their submission* to facilitate the interpretation and replication of experiments. Authors can upload supporting documentation to indicate the use of appropriate reporting guidelines for health-related research (see [EQUATOR Network](http://www.equator-network.org/%20)), life science research (see the [BioSharing Information Resource](https://biosharing.org/)), or the [ARRIVE guidelines](http://www.plosbiology.org/article/info:doi/10.1371/journal.pbio.1000412) for reporting work involving animal research. Where applicable, authors should refer to any relevant reporting standards documents in this form.

If you have any questions, please consult our Journal Policies and/or contact us: [editorial@elifesciences.org](mailto:editorial@elifesciences.org).

**Sample-size estimation**

- You should state whether an appropriate sample size was computed when the study was being designed
- You should state the statistical method of sample size computation and any required assumptions
- If no explicit power analysis was used, you should describe how you decided what sample (replicate) size (number) to use

Please outline where this information can be found within the submission (e.g., sections or figure legends), or explain why this information doesn’t apply to your submission:

Sample sizes were determined empirically based on similar previous measurements of body weight, food intake, and energy expenditure. The final sample sizes were also affected by acquisition of specific genotypes from transgenic mouse breeding. Specific sample size for each experiment can be found in corresponding figure graphs and legends.

**Replicates**

- You should report how often each experiment was performed
- You should include a definition of biological versus technical replication
- The data obtained should be provided and sufficient information should be provided to indicate the number of independent biological and/or technical replicates
- If you encountered any outliers, you should describe how these were handled
- Criteria for exclusion/inclusion of data should be clearly stated
- High-throughput sequence data should be uploaded before submission, with a private link for reviewers provided (these are available from both GEO and ArrayExpress)

Please outline where this information can be found within the submission (e.g., sections or figure legends), or explain why this information doesn’t apply to your submission:

**1. Electrophysiology**

1) Puff application of leptin (figure 1): n=23 for recordings without synaptic blockers; n=25 for recordings with synaptic blockers.

2) Bath application of leptin (figure 1 – figure supplement 1): n=12 for recordings without synaptic blockers.

**2. POA LepRb stimulation**

1) Food intake measurement with BioDAQ (figure 2): n=8; PBS injection data are averages of 3 injections on different days while leptin injection data are averages of 2 injections on different days for each concentration.

2) Indirect calorimetry with TSE (figure 3): n=8; PBS data are from 1 injection and leptin data are averages of 2 separate injections for each temperature condition. This information can be found in *Materials and methods* under ‘POA leptin injection’.

**3. POA LepRb knockdown**

1) Regular chow feeding (figure 4): control group (AAV-GFP) – 4 males and 2 females; knockdown group (AAV-GFP:Cre) – 5 males and 4 females. Body weight and food intake were measured weekly, and energy expenditure was measured at 4 different time points. For each time point, 24hr energy expenditure was averaged over 3 days.

2) High fat diet feeding (figure 5): control group (AAV-GFP) – 6 males and 9 females; knockdown group (AAV-GFP:Cre) – 6 males and 10 females. We measured body weight and food intake weekly and body composition every 3 weeks. Energy expenditure was measured at 3 different time points. 24hr energy expenditure was averaged over 2 or 3 days.

This information can be found in *Materials and methods* under ‘Selective *LepRb* KD in the POA’ and figure 4 and 5 legends.

In all experiments, no outlier was excluded.

**Statistical reporting**

- Statistical analysis methods should be described and justified
- Raw data should be presented in figures whenever informative to do so (typically when N per group is less than 10)
- For each experiment, you should identify the statistical tests used, exact values of N, definitions of center, methods of multiple test correction, and dispersion and precision measures (e.g., mean, median, SD, SEM, confidence intervals; and, for the major substantive results, a measure of effect size (e.g., Pearson's r, Cohen's d)
- Report exact p-values wherever possible alongside the summary statistics and 95% confidence intervals. These should be reported for all key questions and not only when the p-value is less than 0.05.

Please outline where this information can be found within the submission (e.g., sections or figure legends), or explain why this information doesn’t apply to your submission:

**1. POA LepRb stimulation**

1) Food intake measurement with BioDAQ (figure 2): repeated measures ANOVA was used to test the effect of treatment and hours. Bonferroni correction was used for multiple comparisons.

2) Indirect calorimetry with TSE (figure 3): energy expenditure and food intake were compared between treatments in each temperature condition by pair *t*-test.

**2. POA LepRb knockdown**

1) Regular chow feeding (figure 4): repeated measures ANOVA was used to test group (virus type) and time (week) effects on body weight, food intake and energy expenditure. Multiple comparisons were corrected by Bonferroni correction. To test the effect of energy state (diet) on energy expenditure, one-way ANOVA was used (figure 4G). Leptin’s effect on fasting-induced hypometabolism was tested by repeated measures ANOVA (group x treatment).

2) High fat diet feeding (figure 5): repeated measures ANOVA was used to test group and time effects on body weight, food intake and energy expenditure. Multiple comparisons were corrected by Bonferroni correction. Simple group comparisons on plasma T3, energy expenditure, BAT gene expression, and female reproduction were tested by independent *t*-test. Correlations between the POA pSTAT3-positive cell number and various parameters were tested by Pearson correlation.

Detailed information on statistical analysis can be found in corresponding figure legends.

(For large datasets, or papers with a very large number of statistical tests, you may upload a single table file with tests, Ns, etc., with reference to sections in the manuscript.)

**Group allocation**

- Indicate how samples were allocated into experimental groups (in the case of clinical studies, please specify allocation to treatment method); if randomization was used, please also state if restricted randomization was applied
- Indicate if masking was used during group allocation, data collection and/or data analysis

Please outline where this information can be found within the submission (e.g., sections or figure legends), or explain why this information doesn’t apply to your submission:

In POA LepRb stimulation study, each mouse received all treatments.

In POA LepRb knockdown study, mice were randomly allocated into experimental groups based on age and body weight such that the mean body weights of each group were as close to each other while age distributions between groups are similar. For high fat diet feeding, two cohorts of mice were used based on their age. Each cohort is a mixture of mice from both groups and two cohorts were separated by about two months. Weekly body weight and food intake were measured by a staff who was oblivious of group assignment.

**Additional data files (“source data”)**

- We encourage you to upload relevant additional data files, such as numerical data that are represented as a graph in a figure, or as a summary table
- Where provided, these should be in the most useful format, and they can be uploaded as “Source data” files linked to a main figure or table
- Include model definition files including the full list of parameters used
- Include code used for data analysis (e.g., R, MatLab)
- Avoid stating that data files are “available upon request”

Please indicate the figures or tables for which source data files have been provided:

No additional data files have been provided.
